# The Cost of Living Index as a Primary Driver of Homelessness in the United States: A Cross-State Analysis

**DOI:** 10.7759/cureus.46975

**Published:** 2023-10-13

**Authors:** Thomas F Heston

**Affiliations:** 1 Medical Education and Clinical Sciences, Washington State University, Spokane, USA; 2 Family Medicine, University of Washington, Spokane, USA

**Keywords:** cost of living index, poverty rate, multivariate regression, predictive variables, homelessness

## Abstract

Background: Homelessness persists as a critical global issue despite myriad interventions. This study analyzed state-level differences in homelessness rates across the United States to identify influential societal factors to help guide resource prioritization.

Methods: Homelessness rates for 50 states and Washington, DC, were compared using the most recent data from 2020 to 2023. Twenty-five variables representing potential socioeconomic and health contributors were examined. The correlation between these variables and the homelessness rate was calculated. Decision trees and regression models were also utilized to identify the most significant factors contributing to homelessness.

Results: Homelessness rates were strongly correlated with the cost of living index (COLI), housing costs, transportation costs, grocery costs, and the cigarette excise tax rate (all: P < 0.001). An inverse relationship was observed between opioid prescription rates and homelessness, with increased opioid prescribing associated with decreased homelessness (P < 0.001). Due to collinearity, the combined cost of living index was used for modeling instead of its individual components. Decision tree and regression models identified the cost of living index as the strongest contributor to homelessness, with unemployment, taxes, binge drinking rates, and opioid prescription rates emerging as important factors.

Conclusion: This state-level analysis revealed the cost of living index as the primary driver of homelessness rates. Unemployment, poverty, and binge drinking were also contributing factors. An unexpected negative correlation was found between opioid prescription rates and homelessness. These findings can help guide resource allocation to address homelessness through targeted interventions.

## Introduction

Homelessness remains a pervasive global issue. In the United States, over 500,000 people were estimated to be without adequate housing on any given night in 2020. Europe reported even higher figures, exceeding 700,000. In these regions, the estimated per capita rate of homelessness is approximately 0.2%, equating to one in every 500 individuals [1]. Additionally, mobile populations such as people seeking refuge and migrants, who comprise about 3%-5% of the global populace, confront similar challenges [2].

One of the major issues contributing to homelessness is the rising cost of housing. Housing accounted for a fifth of inflation in 2022 in the United States. However, by March 2023, the housing inflation rate rose to 2.6 percentage points, accounting for half of the annual consumer price index inflation [3]. With rental prices rising, even minor changes are estimated to affect homelessness substantially [4].

The health consequences of homelessness are severe. Estimates suggest a lifespan reduction of 5-10 years for homeless individuals [5]. Furthermore, age-adjusted mortality rates for the homeless in New York shelters are two to four times higher than the general population [6]. Homeless individuals have higher rates of chronic health issues, mental health disorders, and substance abuse [7,8]. Homeless children have elevated developmental delays and abuse rates but reduced access to social services to address these issues [9].

Various intervention strategies have yielded some success. When augmented with social services, permanent supportive housing improves long-term stability [10]. Offering immediate housing without preconditions has been shown effective in achieving initial housing stability. Still, participants may be more prone to incarceration and are less prepared for independent living upon discharge [11].

Despite these programmatic successes, homelessness rates have risen, underscoring the need for more effective interventions. This study aims to identify societal-level factors influencing homelessness by examining variations in rates across all 50 US states and Washington, DC. The insights obtained will help tailor community-specific solutions, enhancing the effectiveness of homelessness intervention programs.

The initial draft of this article was previously posted to the medRxiv preprint server on September 21, 2023 [12].

## Materials and methods

Sample

State-level data were collected for the 50 US states and Washington, DC. The most recent data available as of September 2023 were obtained. Variables examined included homelessness, unemployment, cost of living index (COLI), grocery cost index, housing cost index, utility cost index, transportation cost index, health cost index, poverty rate, per capita real gross domestic product (GDP), drug overdose mortality, median household income, incarceration rate, gasoline price, Gini coefficient, average state and local taxes, percentage of income spent on housing (renters), binge drinking prevalence, opioid prescriptions per capita, smoking prevalence, high school graduation rate, cigarette tax, alcohol consumption per capita, 2020 presidential election results, state population, incarceration rate, and sanctuary status. When possible, data were retrieved from official government sources. Ethical review board approval was not required as this study analyzed existing public data. No human or animal research was performed. Data collected with detailed source information for this analysis is publicly available in the Zenodo repository [13].

Statistical analysis

Both conventional statistical tests and machine learning techniques were utilized to examine associations with homelessness. The Statistical Package for the Social Sciences (SPSS) version 29 (IBM SPSS Statistics, Armonk, NY) was utilized for all analyses, except for random forest, which was done using Python. Normality was assessed using the Shapiro-Wilk test. Spearman correlation with Bonferroni correction evaluated associations between variables. Machine learning techniques, including exhaustive Chi-square Automatic Interaction Detection (CHAID), classification and regression trees (CRT), and random forest regression, were utilized to identify variables associated with homelessness rates. Exhaustive CHAID is a segmentation modeling approach that identifies groups using chi-square tests. CRT builds decision trees for classification or regression objectives using recursive binary splitting. Random forest is an ensemble technique aggregating results from many decision trees built using random subsets of variables and samples. These were followed by automatic linear modeling and backward linear regression, manually removing nonsignificant variables. Collinearity tolerances were evaluated for multicollinearity, and autocorrelation was evaluated with Durbin-Watson testing. A test of proportions compared binary categorical variables (political party and sanctuary status) with homelessness.

## Results

**Figure 1:**
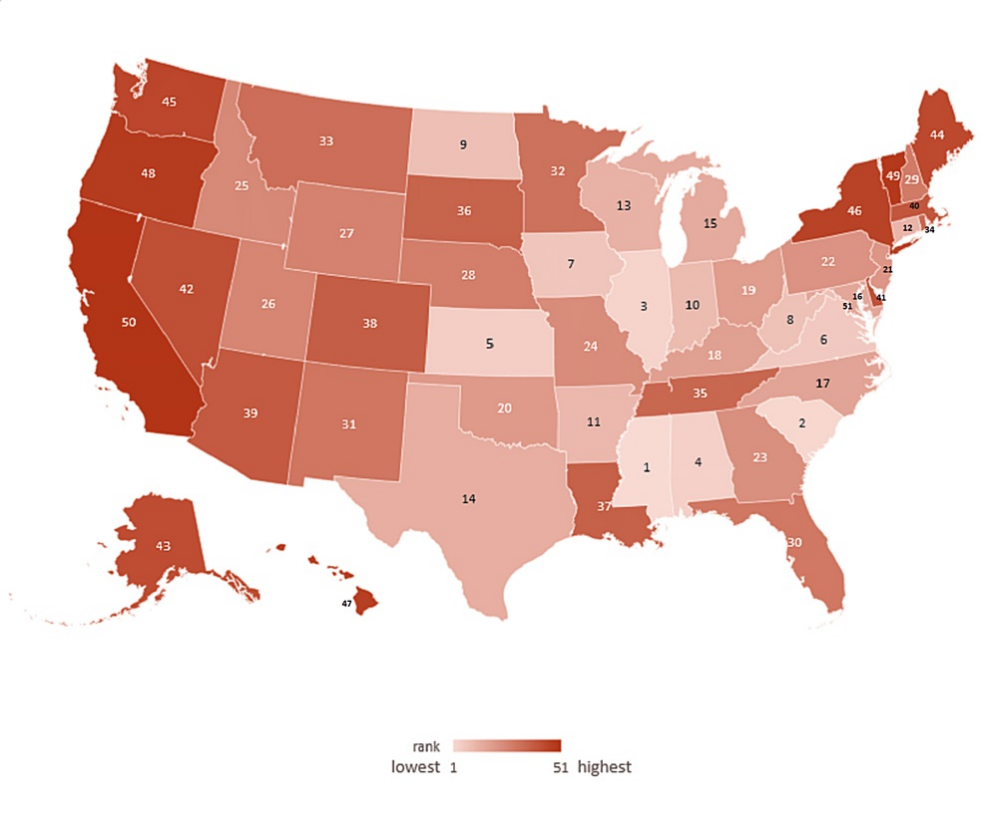
Heatmap of homelessness rates in 2022 ranked for 50 US states and Washington, DC Image credits: Thomas F. Heston (author)

Homelessness rates per 10,000 people in 2022 ranged from a low of 4.07 in Mississippi to 65.8 in Washington, DC, with an average of 16.5 (Figure 1) [14].

Data were not normally distributed per the Shapiro-Wilk test (P < 0.05). Transformation attempts were unsuccessful in normalizing distributions, so nonparametric tests were the primary means of statistical analysis.

After Bonferroni correction for multiple testing, significant correlations with homelessness rates were found for the housing cost index, cost of living index, transportation cost index, grocery cost index, cigarette excise tax, and opioid prescriptions per capita (all: P < 0.001). Higher opioid prescription rates were associated with lower homelessness. Some correlations were significant by univariate P-value analysis, but not significant when the Bonferroni correction for multiple testing was applied. Specifically, states that voted Republican in the previous presidential election (2020) had less homelessness; states with a higher overall tax burden had less homelessness; a higher state income was associated with more homelessness; a higher overall healthcare cost index was associated with more homelessness; a higher housing burden, in which greater than 30% of income was devoted to housing costs, was associated with greater homelessness; and finally, a higher state gross domestic product was associated with more homelessness. The Bonferroni corrected significance was applied because the multiple correlations increased the risk of false positives (Table 1).

**Table 1:** Correlation coefficients with homelessness The most significant correlations with homelessness were related to financial strain.

Variable	Coefficient	P-value	Bonferroni significance
Housing cost index	0.721	<0.001	Significant
Cost of living index	0.683	<0.001	Significant
Transportation cost index	0.628	<0.001	Significant
Grocery cost index	0.618	<0.001	Significant
Cigarette excise tax	0.499	<0.001	Significant
Opioid prescriptions per capita	-0.464	<0.001	Significant
Voted Republican last presidential election	-0.429	0.002	Nonsignificant
Overall tax burden	-0.391	0.004	Nonsignificant
Income	0.381	0.006	Nonsignificant
Healthcare cost index	0.333	0.017	Nonsignificant
Housing burden	0.317	0.023	Nonsignificant
State gross domestic product	0.209	0.027	Nonsignificant
Incarceration rate	-0.262	0.063	Nonsignificant
Alcohol binge rate	0.221	0.119	Nonsignificant
High school graduation rate	0.221	0.119	Nonsignificant
Unemployment	0.219	0.122	Nonsignificant
Population	-0.19	0.181	Nonsignificant
Alcohol consumption rate	0.181	0.204	Nonsignificant
Poverty	-0.165	0.248	Nonsignificant
Sanctuary status	0.154	0.282	Nonsignificant
Utilities cost index	0.144	0.313	Nonsignificant
Gasoline price per gallon	0.13	0.363	Nonsignificant
Drug overdose mortality rate	-0.073	0.608	Nonsignificant
Smoking rate	0.049	0.733	Nonsignificant
Gini coefficient	-0.044	0.76	Nonsignificant

Housing, transportation, and groceries were the key drivers of the cost of living index. Due to multicollinearity, these were consolidated into the overall cost of living index for the classification and regression modeling analyses.

Decision tree-based methods were then utilized to help further clarify and identify significant factors associated with homelessness. The exhaustive CHAID analysis was done with five parent and five child nodes, with the Bonferroni correction not enforced during model building. The resultant F values were normalized to add up to 100% to determine relative importance. This identified the cost of living index (0.642), state and local taxes (0.179), alcohol binging per capita (0.098), and opioid prescriptions per capita (0.081) as the most important factors, with a risk estimate of 47.8 and a standard error of 14.7.

CRT analysis was then done with manual weaning of factors to produce the strongest model based on its risk estimate and the importance factors normalized to add up to 100%. Using this method, the factors identified as significant were the cost of living index (0.502), unemployment rate (0.362), and poverty (0.136).

A random forest analysis was then done. After a step-by-step process involving removing the least important factors, the best model consisted of the cost of living index (importance 0.642), alcohol binge rate (0.122), unemployment (0.091), taxes (0.085), and poverty (0.060). This model explained 65% of the variation in homelessness with an R-squared value of 0.655. Adding in the other factors did not improve this model. Notably, adding back in the opioid prescription rate did not improve the model but slightly decreased the R-squared value to 0.632.

SPSS Automatic Linear Modeling was then done using its built-in automatic weaning of insignificant factors. The resultant model was reviewed, and any remaining insignificant factors were removed. Using this method, the factors identified as significant were the cost of living index (importance 0.722, P < 0.001), unemployment (0.120, P = 0.003), alcohol binge rate (0.089, P = 0.011), and taxes (0.069, P = 0.023). Finally, a linear regression model was done using backward processing and manual removal of low correlates. Importance was based on the standardized beta coefficient. The final model, after backward elimination, identified as significant the cost of living index (importance 0.564, P < 0.001), unemployment (0.245, P = 0.004), and alcohol binge rate (0.192, P = 0.018) with an adjusted R-squared value of 0.643. Collinearity tolerances were 0.871, 0.835, and 0.880, respectively, consistent with no significant multicollinearity. Durbin-Watson was 1.956, consistent with no autocorrelation. The adjusted R square for the model was 0.643, explaining 64% of the variation in homelessness. The unstandardized beta for COLI was 0.431, estimating that a decrease of 10% in COLI would result in a 4.3% decrease in homelessness.

Significant factors contributing to homelessness identified by the various models are summarized in Table 2, along with the average overall importance.

**Table 2:** Relative importance of factors contributing to homelessness In all models, the cost of living index was the most important factor contributing to homelessness. CHAID: Chi-square Automatic Interaction Detection, CRT: classification and regression trees

Variable	Exhaustive CHAID	CRT	Random forest	Automatic linear modeling	Linear regression	Overall
Cost of living index	64.2%	50.2%	64.2%	72.2%	56.4%	61.4%
Unemployment	0%	36.2%	9.1%	12%	24.5%	16.4%
Alcohol binge rate	9.8%	0%	12.2%	8.9%	19.2%	10%
Taxes	17.9%	0%	8.5%	6.9%	0%	6.7%
Poverty	0%	13.6%	6%	0%	0%	3.9%
Opioid prescriptions	8.1%	0%	0%	0%	0%	1.6%
Total	100%	100%	100%	100%	100%	100%

The cost of living index was the most important factor in all five models and the most important overall, contributing 61.4% to the combined model. Unemployment was an important factor in four of the five models and overall contributed 16.4%. The alcohol binge rate was also identified as an important factor in four of the five models, with an overall contribution of 10%. Taxes were identified as important in three models, poverty in two, and the opioid prescription rate in one.

The cigarette excise tax was significantly correlated with homelessness by Spearman correlation. However, none of the three classifications and two regression models identified it as a significant contributor.

## Discussion

This study identified the cost of living index, primarily driven by housing, transportation, and grocery costs, as the predominant factor associated with state-level homelessness rates. Across all models, the cost index was weighted 61.4% in importance as a contributing factor to homelessness. Unemployment, alcohol consumption, taxes, opioid prescription rates, and poverty also emerged as significant contributors. These findings highlight the multifactorial determinants of homelessness, with economic housing factors playing the predominant role.

Our data show that the cost of living index was consistently the most influential factor affecting homelessness. The primary component affecting the cost of living index was housing costs. This aligns with previous research demonstrating that rent costs are the most significant predictor of homelessness [15]. Thus, policies that enhance rental housing affordability, such as rent control, public housing, renal subsidies, and housing vouchers, may help reduce homelessness. Based on our regression models, a 10% reduction in housing costs is estimated to lower homelessness rates by around 4.5% across states. Although this ecological estimate has limitations, it suggests even modest gains in affordability could meaningfully impact homelessness. Also, one study found that reducing housing costs helps mitigate homelessness [16], and another found a rapid rise in homelessness in communities where people spend more than a third of their income on rent [17]. While more research is needed to identify the most effective housing policies, interventions that reduce housing costs will likely be the most impactful.

Unemployment was also a major factor associated with homelessness rates, second only to the cost of living index. This confirms previous research showing that job loss increases the risk of homelessness [18]. Vocational training may help address this issue by decreasing recidivism after incarceration [19], reducing the need for mental health services [20], and decreasing unhealthy behaviors such as excessive alcohol use [21]. However, while unemployment was an important factor, the overall cost of living index had a stronger association with homelessness. This suggests that vocational training alone cannot fully address homelessness if structural economic factors such as lack of affordable housing persist. This suggests that while vocational training may help, a comprehensive approach requires addressing both unemployment and the overall cost of living.

Our analysis found states with higher binge drinking rates tended to have higher homelessness rates. This aligns with prior evidence showing alcohol use disorders are disproportionately common in homeless populations, with approximately 38% of homeless adults meeting the criteria for alcohol abuse or dependence [8]. Another study found that hazardous drinking increased the risk of subsequent homelessness by about 40% [22]. Binge drinking may also directly worsen housing instability through its associations with a myriad of adverse health and economic consequences [23]. Underutilization of substance abuse services may also increase recurrent homelessness [24]. Overall, our findings and previous research indicate that binge drinking and homelessness are significantly intertwined, suggesting that effectively mitigating one phenomenon may require concurrently addressing the other as well as root causes such as past trauma or mental health issues.

It was found that the tax burden and poverty rate were associated with increased homelessness. The relationship with tax burden is unclear and has not been well studied. While taxes can help pay for housing subsidies, property taxes increase housing costs. The solution to this is not clear. For example, one study found that increasing the Earned Income Tax Credit reduced housing cost burdens but did not reduce homelessness [25]. On the other hand, poverty is a significant risk factor for homelessness [26]. Yet the rise in housing costs continues to outpace increases in income [27].

Unexpectedly, higher opioid prescription rates correlated with lower homelessness, contrasting with previous studies [28]. This warrants a deeper investigation into whether restrictive opioid policies are unintentionally displacing chronically ill patients toward dangerous street drugs and housing insecurity. Integrating harm reduction approaches into housing programs may help mitigate overdose risks in this population.

Although our study focused on homelessness, this finding of increased opioid prescribing being associated with lower homelessness is plausible and supported by previous research. Studies have shown that a singular focus on restricting prescription opioids may have the unintended side effect of a compensatory increase in illicit heroin use, overdosing, and death [29,30]. As patients lose stable access to prescription opioids to manage chronic conditions, there is a concern that patients will turn to illicit substances [31]. Furthermore, abruptly discontinuing opioids in dependent patients can precipitate withdrawal, depression, anxiety, and suicidality [32]. Decreased access to prescription opioids resulting in a shift to illicit opioids and the psychological effects of withdrawal may hinder patients' ability to maintain employment and housing. Hence, our results imply that restrictive opioid policies could inadvertently worsen housing insecurity. Additionally, incorporating medication-assisted treatment into supportive housing programs may help maintain housing stability for those with opioid use disorder. Further longitudinal research on this association is warranted to understand better the impact of opioid prescribing policies on homelessness risk over time.

This study possessed inherent limitations. The cross-sectional ecological design using group-level data restricted causal inference and omitted time-dependent effects. Although multiple analytical techniques were leveraged, the potential for unmeasured confounding variables remained. The reliance on secondary data introduced possible inaccuracies or biases. The generalizability of our models required context-specific interpretation given state-level heterogeneity. In addition, univariate correlations did not always agree with the models utilized to identify significant factors. This finding is not unexpected, as correlation coefficients look at linear one-to-one relationships and do not consider nonlinear relationships or the interplay of variables upon one another. Thus, the models taking into account nonlinear relationships and the interaction between variables were considered to be a more accurate and meaningful analysis of the data.

Nonetheless, these findings can help guide resource allocation and policy decisions. Our results strongly support affordable housing interventions, as this was the predominant factor associated with lower homelessness rates. Decreasing housing, transportation, and nutritional assistance costs should be prioritized. Expanding job creation and integrated addiction treatment access could help mitigate homelessness and secondary consequences such as overdoses. A collaborative, multifaceted response will ultimately be essential to address this persistent public health crisis.

## Conclusions

This study identified the cost of living index, mainly housing, as the predominant factor associated with state-level homelessness rates, underscoring economic stability as a priority. Unemployment, binge drinking, and poverty also contributed significantly. Lower opioid prescription rates were associated with increased homelessness, suggesting the need for further research into whether excessively restricting prescription opioids may have unintended consequences such as increased use of illicit opioids and housing insecurity. Limitations such as the ecological design restrict causal inference, yet the insights may aid future research and policy. The results strongly support housing-focused interventions aimed at increasing affordability and access and addressing income insecurity through job creation, transportation access, and nutritional assistance. Ultimately, collaborative, multifaceted efforts are needed to alleviate homelessness. This study highlights actionable targets to reduce homelessness and its associated health consequences.

## References

[1] Henry M, de Sousa T, Roddey C, Gayen S, Bednar Bednar, Abt Associates (2021). The 2020 Annual Homeless Assessment Report (AHAR) to Congress. Development.

[2] Crawford G, Connor E, McCausland K, Reeves K, Blackford K (2022). Public health interventions to address housing and mental health amongst migrants from culturally and linguistically diverse backgrounds living in high-income countries: a scoping review. Int J Environ Res Public Health.

[3] (2023). White House: An update on housing inflation in the consumer price index. https://www.whitehouse.gov/cea/written-materials/2023/04/27/update-on-housing-inflation-in-cpi/.

[4] Quigley JM, Raphael S (2001). The economics of homelessness: the evidence from North America. Eur J Hous Pol.

[5] Funk AM, Greene RN, Dill K, Valvassori P (2022). The impact of homelessness on mortality of individuals living in the United States: a systematic review of the literature. J Health Care Poor Underserved.

[6] Barrow SM, Herman DB, Córdova P, Struening EL (1999). Mortality among homeless shelter residents in New York City. Am J Public Health.

[7] Aldridge RW, Story A, Hwang SW (2018). Morbidity and mortality in homeless individuals, prisoners, sex workers, and individuals with substance use disorders in high-income countries: a systematic review and meta-analysis. Lancet.

[8] Fazel S, Khosla V, Doll H, Geddes J (2008). The prevalence of mental disorders among the homeless in western countries: systematic review and meta-regression analysis. PLoS Med.

[9] Vostanis P, Grattan E, Cumella S, Winchester C (1997). Psychosocial functioning of homeless children. J Am Acad Child Adolesc Psychiatry.

[10] Aubry T, Bloch G, Brcic V (2020). Effectiveness of permanent supportive housing and income assistance interventions for homeless individuals in high-income countries: a systematic review. Lancet Public Health.

[11] Hall G, Walters S, Gould H, Lim S (2020). Housing versus treatment first for supportive housing participants with substance use disorders: a comparison of housing and public service use outcomes. Subst Abus.

[12] Heston TF (2023). The cost of living index as a primary driver of homelessness: a cross-state analysis. medRxiv.

[13] Heston TF (2023). Dataset for the cost of living index as a primary driver of homelessness. Zenodo.

[14] de Sousa T, Andrichik A, Cuellar M, Marson J, Prestera E, Rush K, Abt Associates (2022). The 2022 Annual Homelessness Assessment Report (AHAR) to Congress. https://www.huduser.gov/portal/sites/default/files/pdf/2022-AHAR-Part-1.pdf.

[15] Byrne T, Munley EA, Fargo JD, Montgomery AE, Culhane DP (2013). New perspectives on community-level determinants of homelessness. J Urban Aff.

[16] Elliott M, Krivo LJ (1991). Structural determinants of homelessness in the united states. Soc Probl.

[17] (2023). Zillow Research: Homelessness rises faster where rent exceeds a third of income. https://www.zillow.com/research/homelessness-rent-affordability-22247/.

[18] Burke C, Johnson EE, Bourgault C, Borgia M, O'Toole TP (2013). Losing work: regional unemployment and its effect on homeless demographic characteristics, needs, and health care. J Health Care Poor Underserved.

[19] Newton D, Day A, Giles M, Wodak J, Graffam J, Baldry E (2018). The impact of vocational education and training programs on recidivism: a systematic review of current experimental evidence. Int J Offender Ther Comp Criminol.

[20] Jackson Y, Kelland J, Cosco TD, McNeil DC, Reddon JR (2009). Nonvocational outcomes of vocational rehabilitation: reduction in health services utilization. Work.

[21] Swahn MH, Buchongo P, Kasirye R (2018). Risky behaviors of youth living in the slums of Kampala: a closer examination of youth participating in vocational training programs. Vulnerable Child Youth Stud.

[22] Ghose T, Fiellin DA, Gordon AJ (2013). Hazardous drinking and its association with homelessness among veterans in care. Drug Alcohol Depend.

[23] Rehm J, Mathers C, Popova S, Thavorncharoensap M, Teerawattananon Y, Patra J (2009). Global burden of disease and injury and economic cost attributable to alcohol use and alcohol-use disorders. Lancet.

[24] McQuistion HL, Gorroochurn P, Hsu E, Caton CL (2014). Risk factors associated with recurrent homelessness after a first homeless episode. Community Ment Health J.

[25] Pilkauskas N, Michelmore K (2019). The effect of the earned income tax credit on housing and living arrangements. Demography.

[26] Giano Z, Williams A, Hankey C, Merrill R, Lisnic R, Herring A (2020). Forty years of research on predictors of homelessness. Community Ment Health J.

[27] (2023). National Low Income Housing Coalition: Out of reach: The high cost of housing: How much do you need to earn to afford a modest apartment in your state?. https://nlihc.org/oor.

[28] Cano M, Oh S (2023). State-level homelessness and drug overdose mortality: evidence from US panel data. Drug Alcohol Depend.

[29] Alpert A, Powell D, Pacula RL (2018). Supply-side drug policy in the presence of substitutes: evidence from the introduction of abuse-deterrent opioids. Am Econ J Econ Policy.

[30] Evans WN, Lieber EM, Power P (2019). How the reformulation of oxycontin ignited the heroin epidemic. The review of Economics and statistics.

[31] Lagisetty PA, Healy N, Garpestad C, Jannausch M, Tipirneni R, Bohnert AS (2019). Access to primary care clinics for patients with chronic pain receiving opioids. JAMA Netw Open.

[32] Frank JW, Lovejoy TI, Becker WC (2017). Patient outcomes in dose reduction or discontinuation of long-term opioid therapy: a systematic review. Ann Intern Med.

